# Efficacy of extracranial stereotactic body radiation therapy (SBRT) added to standard treatment in patients with solid tumors (breast, prostate and non-small cell lung cancer) with up to 3 bone-only metastases: study protocol for a randomised phase III trial (STEREO-OS)

**DOI:** 10.1186/s12885-021-07828-2

**Published:** 2021-02-04

**Authors:** Sébastien Thureau, Vincent Marchesi, Marie-Hélène Vieillard, Lionel Perrier, Albert Lisbona, Marianne Leheurteur, Jean Tredaniel, Stéphane Culine, Bernard Dubray, Naïma Bonnet, Bernard Asselain, Julia Salleron, Jean-Christophe Faivre

**Affiliations:** 1 Radiation Oncology & Medical Physics Department, Henri-Becquerel Comprehensive Cancer Center, rue d’Amiens, F-76 000 Rouen, France; 2 EA4108 QuantIf Litis, University of Rouen, 22 boulevard Gambetta, 76000 Rouen, France; 3 Academic Radiation Oncology & Brachytherapy Department, Lorraine Institute of Cancerology - Alexis-Vautrin Comprehensive Cancer Center, 6 avenue de Bourgogne, 54519 Vandœuvre-lès-Nancy, France; 4 Rheumatology Department, University Hospital of Lille, 2 avenue Oscar Lambret, 59 000 Lille, France; 5 UMR CNRS 5824, Léon Bérard Comprehensive Cancer Center, 28 rue laennec, 69 373 Lyon, France; 6 Academic Radiation Oncology & Brachytherapy Department, Institut de Cancérologie de l’Ouest – René Gauducheau Comprehensive Cancer Center, Boulevard Professeur Jacques Monod, 44805 Saint-Herblain, France; 7 Medical Oncology Department, Henri-Becquerel Comprehensive Cancer Center, rue d’Amiens, 76000 Rouen, France; 8 Pneumology Department, University Hospital of Paris (Groupe hospitalier Paris Saint-Joseph), 185 Rue Raymond Losserand, 75014 Paris, France; 9 Medical Oncology Department, University Hospital of Paris (Saint-Louis Hospital), 1 avenue Claude Vellefaux, 75010 Paris, France; 10 Paris Diderot University, 16 rue Huchard, 75018 Paris, France; 11 Unicancer, 101, rue de Tolbiac, F-75654 Paris, France; 12 Biostatistics Department, Institut de Cancérologie de Lorraine - Alexis-Vautrin Comprehensive Cancer Center, 6 avenue de Bourgogne, F-54519 Vandœuvre-lès-Nancy, France

**Keywords:** MeSH: Oligometastases, Bone metastases, Stereotactic radiotherapy, Neoplasm metastasis, Radiosurgery, Lung neoplasms, Prostatic neoplasms, Breast neoplasms

## Abstract

**Background:**

Stereotactic Body Radiation Therapy (SBRT) is an innovative modality based on high precision planning and delivery. Cancer with bone metastases and oligometastases are associated with an intermediate or good prognosis. We assume that prolonged survival rates would be achieved if both the primary tumor and metastases are controlled by local treatment. Our purpose is to demonstrate, via a multicenter randomized phase III trial, that local treatment of metastatic sites with curative intent with SBRT associated of systemic standard of care treatment would improve the progression-free survival in patients with solid tumor (breast, prostate and non-small cell lung cancer) with up to 3 bone-only metastases compared to patients who received systemic standard of care treatment alone.

**Methods:**

This is an open-labeled randomized superiority multicenter phase III trial. Patients with up to 3 bone-only metastases will be randomized in a 1:1 ratio.between Arm A (Experimental group): Standard care of treatment & SBRT to all bone metastases, and Arm B (Control group): standard care of treatment.

For patients receiving SBRT, radiotherapy dose and fractionation depends on the site of the bone metastasis and the proximity to critical normal structures. This study aims to accrue a total of 196 patients within 4 years.

The primary endpoint is progression-free survival at 1 year, and secondary endpoints include Bone progression-free survival; Local control; Cancer-specific survival; Overall survival; Toxicity; Quality of life; Pain score analysis, Cost-utility analysis; Cost-effectiveness analysis and Budget impact analysis.

**Discussion:**

The expected benefit for the patient in the experimental arm is a longer expectancy of life without skeletal recurrence and the discomfort, pain and drastic reduction of mobility and handicap that the lack of local control of bone metastases eventually inflicts.

**Trials registration:**

ClinicalTrials.gov NCT03143322 Registered on May 8th 2017. Ongoing study

## Background

Cancer with bone metastases compared to other metastatic sites is considered as associated with a better prognosis, particularly for breast and prostate cancer despite a significant impact on the patient’s quality of life and autonomy. Many randomized controlled trials have demonstrated the efficacy of first-line palliative conventional radiotherapy for painful bone metastases [1–4]. The advent of novel imaging techniques allows an increasing an early detection and diagnosis of oligometastatic disease. In clinical practice the possibility to identify a few number of lesions, better defined recently by ASTRO, EORTC and ESTRO, it is considered an intermediate phase of tumor spread with limited metastatic capacity [5, 6].

Patients with oligometastatic disease have improved survival compared with those with high- volume metastatic disease. While systemic treatment is the standard of care. Stereotactic radiotherapy is a highly accurate technique initially developed for performing the radiosurgery of lesion in patients for whom it was deemed be to difficult to proceed to classical excision surgery. In the context of patients with oligo-metastatic cancer, high doses of radiation in stereotactic conditions can be safely delivered in bone metastases with increased probability of local control. Local control of bone metastasis is similar to than other localizations with a good control of pain [7, 8] and patients with oligometastasis had a longer survival with better local control for spine metastasis [9] and delay progression, and thereby postpone the need for further systemic treatment [10, 11].

In combination with curative-intent treatment to the primary tumor and systemic therapy, we hypothesize that an improved local control in the bone genuine de novo synchronous or metachronous oligometastatic disease would be associated with an increased Progression-Free Survival (PFS) and Overall Survival (OS).

Two ancillaries studies are associated:

- Biological ancillary study which search for pre-SBRT and follow-up markers (serum CTX and bone ALP) associated with skeletal-related events and bone progression disease.

- Economic ancillary study.

## Methods/design

**Fig. 1:**
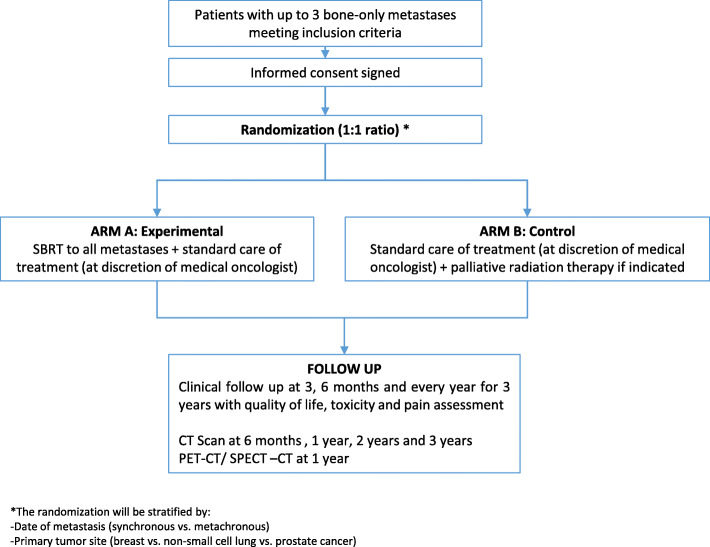
Study Design. Footnotes: SBRT: Stereotactic Body radiation Therapy; CT Scan: Computed tomography Scann; PET: Positron Emission Tomography; SPECT-CT: Single Photon Emission Tomography-Computed Tomography

This is an open-labeled randomized superiority multicentric phase III studyin French academic hospital. Patients will randomized in a 1:1 ratio between (Fig. 1):

- Arm A (experimental group): Standard care of treatment (systemic treatment suitable for primary cancer) and SBRT to the bone metastases.
- Arm B (control group): Standard care of treatment (systemic treatment suitable for primary cancer) alone.

The sample size allows 2 stratification factors at randomization: date of metastases (synchronous vs metachronous) and primary tumor site (prostate vs breast vs lung). All patients will be randomized after completion of the primary tumor treatment for synchronous or metachronous metastasis. The randomization will be minimized by the following stratification factors. Minimization aims to ensure treatment arms are balanced with respect to predefined patient factors as well as for the number of patients in each group.

### Objectives

To assess the impact of SBRT on Progression Free Survival in patients with solid tumor with up to bone-only metastases, compare to patients who received standard of care treatment alone.

#### Primary endpoint

The primary endpoint is the 1-year PFS. Progression Free Survival PFS is defined as the time from randomization until the date of the first objective progression or death (whatever its cause in the absence of progression). Stereo-OS protocol will allow a specific focus on skeletal metastases and is thus expected to bring a clear-cut assessment of the added benefit of stereotactic radiotherapy on PFS.

#### Secondary endpoints

*The secondary endpoints are as follow:*

- PFS at 2 and 3 years defined as the time from randomization until the date of the first objective progression or death
- Bone progression free-survival (BPFS) at 1 year defined as the time from the date of randomization to the date of documented bone progression at the level at the entire skeleton.
- Local control (LC) at 1 year defined as the time from the date of randomization to the date of a documented local event at the level of bone oligometastases presents at randomization.
- Cancer-specific survival (CSS) at 1, 2 and 3 years defined as the time from the date of randomization to the date of documented death from cancer or complication from the treatment.
- Overall survival (OS) at 1, 2 and 3 years defined as the time from the date of randomization to the date of documented death from any cause.
- Acute and late Toxicity at 1, 2 and 3 years assessed by the National Cancer Institute Common Toxicity Criteria (NCI CTC) version 4.0.
- Quality of Life assessed using self-administered questionnaires (EORTC-QLQ-C30, EORTC-QLQ-BM22, and EQ-5D-3L) [12–14].
- Pain score evaluated accordingly to Numeric Scale.
- The Cost- Utility /Cost-Effectiveness / Budget Impact analysis performed on QALYs (Quality-Adjusted Life Years) and ICERs (Incremental Cost-Effectiveness Ratios) calculation based on EQ-5D-3L questionnaire.

### Eligibility criteria

The inclusion criteria are as follow:

- Patients older than 18 years
- Good general condition: WHO performance status ≤1
- Patients with histological proof of breast, non-small cell lung, or prostate cancer
- Absence of co-morbidity contra-indicating radio-chemotherapy or surgery
- Primary tumor accessible to curative-intent treatment (surgery, chemoradiation…) for patients with synchronous metastases (ie randomization after treatment of primitive cancer)
- Patients with between 1 and 3 synchronous or metachronous bone metastases as defined by NaF-PET or conventional SPECT-CT scan and spinal MRI (if necessary) within 6 weeks before randomization
- Bones metastases treatable by SBRT
- Primary cancer considered to be controlled or accessible to curative-intent treatment (surgery, chemoradiation…) in case of locoregional reccurence for metachronous bone oligo-metastatic disease
- Women of childbearing potential and male patients must agree to use adequate contraception for the duration of study participation and up to 3 months following completion of therapy;
- Patients who have received the information sheet, dated and signed the informed consent form
- Affiliated to the social security system

The non-inclusion criteria are as follow:

- Visceral metastases as defined by FDG-PET (or F-Choline-PET for prostate cancer) and cerebral CT or MRI performed
- Previous systemic therapy for metastasis for patients with metachronous metastasis. Prostate and breast cancer patients remain eligible if hormonal treatment was initiated 6 months before enrollment.
- All bone metastasis requiring surgical treatment (spinal cord compression, fracture, …)
- More than 3 bone metastases as defined by NaF-PET or conventional SPECT-CT scan and spinal MRI (if spinal bone metastases on NaF-PET)
- Previous cancer within the 5 years before inclusion (except basal cell carcinoma of the skin, in situ carcinoma of the uterine cervix)
- Previous radiotherapy on bone metastasis (e.g: antalgic radiotherapy)
- Patient enrolled in another therapeutic trial
- Pregnant women or breast feeding mothers,
- Hypersensitivity to the active substance (FDG and NaF or F-Choline for prostate cancer) or to any of the excipients
- Contraindication to MRI (in case of spinal metastases)
- Patients deprived of liberty or placed under the authority of a tutor. Patients with any psychological, familial, sociological or geographical condition potentially hampering compliance with the study protocol and follow-up schedule; those conditions should be discussed with the patient before registration in the trial. Patients unable to understand the purpose of the study (language, etc.).

### Evaluation and randomization

Prior to randomization, a complete history and physical examination is required. Histologically confirmation of malignancy is required, with metastatic disease detected on imaging.

The mechanism of implementing the allocation sequence (stratified randomization) is generated by computer. Clinical research assistant generate the allocation sequence and physician enroll and assign participant to intervention.

Patients must be restaged within 6 weeks before randomization, including brain CT or MRI and CT-TAP. For all patients with a suspicion of spinal bone metastases, a spinal MRI is mandatory.

The patients will also benefit from PET-CT exams before treatment. PET1 F-Choline, NaF and FDG are standard examinations for metastases staging; and PET2 FDG or F-Choline for evaluation. If NaF-PET cannot be done, patients will have a “conventional” biphosphonates SPECT-CT with a tomographic exploration from the vertex to mid-thigh in double or triple field instead.

Patients with prostate cancer are also required to have PSA blood test. A negative pregnancy is required for women of child-bearing age (Table 1).

**Table 1:** Timeline and follow up of the study

	Screening	Randomization	Treatment	Follow up				
				M3	M6	Y1	Y2	Y3
**History & physical**	X		X	X	X	X	X	X
**Brain CT or MRI**	X				X	X	X	X
**TAP CT**	X				X	X	X	X
**FDG PET or F-Choline PET**	X					X		
**PET or SPECT-CT**	X					X		
**Spinal MRI (if spinal bone metastases)**	X				X	X		
**Mammography (breast cancer)**	X				X	X	X	X
**Histological confirmation**	X							
**Quality of Life**	X		X	X	X	X	X	X
**Toxicity Assessment**			X	X	X	X	X	X
**Pain Assessment**	X		X	X	X	X	X	X
**Economic Study**			X	X	X	X	X	X
**sCTX/ bALP Evaluation**	X		X	X	X	X		

*MRI* Magnetic Resonance Imaging; *TAP-CT* Computed tomography thorax-abdomen-pelvis; *FDG-PET* Fluorodesoxyglucose-Positron Emission Tomography; *F-Choline-PET* Fluor-Choline-Positron Emission Tomography; *PET* Positron Emission Tomography; *SPECT-CT* Single Photon Emission Tomography-Computed Tomography; *sCTX* serum C-Telopeptide cross-link of type 1 collagen; *bALP* bone ALkaline Phosphatase

The present research project will be developed for 48 months, during which specific milestones will be achieved and documented (Table 1).

### Interventions/treatments

#### Arm A

In case of SBRT, the acceptable regimens are 35 Gy / 7 fractions / 3 fractions per week or 27 Gy / 3 fractions / 3 fractions per week. An interval of at least 24 h should be kept between two consecutive fractions. Treatment planning and delivery will be performed using dedicated system. Strict quality assurance protocols will be implemented to ensure the accuracy in dose delivery.

All patients in Arm A will undergo planning CT simulation. CT simulation must be performed in the treatment position with adequate immobilization devices and the fiducials must be placed for stereotactic targeting if necessary. The use of intravenous contrast is recommended. Axial reconstructions will be required with slice thickness of around 1 mm. MRI, and PET if needed will be merged with CT simulation at the target volume on the simulation CT.

The Gross Tumor Volume (GTV) corresponds to the tumor volume visible on the simulation CT taking into account the information provided by the clinical examination and the other imaging modalities. (MRI is mandatory for spinal metastasis and experimental arm).

The Clinical Target Volume (CTV) corresponds to the macroscopic spread. Its limits are defined by Cox et al. for spine metastases or by CTV = GTV + 5 mm for the other bones [15].

The PTV is obtained by adding a margin around the CTV to take into account the set-up uncertainty during radiotherapy delivery with PTV = CTV + 2 mm. This margin can be reduced to 0 mm close to the spinal cord. Depending on the SBRT equipment, the beam aperture can be set to 2–3 mm beyond the CTV to provide adequate dose coverage. This margin can be reduced to 0–1 mm close to the spinal cord.

Prescription must be defined on 80% isodose or higher. At least 90% of the PTV should receive the prescribed dose. A coverage of < 90% of the PTV will be considered as Acceptable Deviation, and coverage of < 80% of the target volume as an unacceptable deviation. At least 90% of the GTV should receive the prescribed dose. A coverage of < 90% of the GTV will be considered as Acceptable Deviation, and coverage of < 80% of the target volume as an unacceptable deviation.

The organs at risk (OAR) of complication include all normal tissues located in the vicinity of the target or in the beams path. Specific dose-volumes constraints, defined by Timmermann, have to be respected to avoid unacceptable toxicity [16]. In the Experimental group with SBRT, all relevant OARs must be delineated. As for the spinal cord, two volumes have to be delineated: A “conventional” volume is delineated on the simulation CT after registration of MRI (T2-weighted and T1-weighted with contrast). The contour should be drawn at least 10 cm above and below the CTV, as recommended by NRG Oncology protocols. A “partial” volume is specific to the present study (Table 2). The contour drawn as above (“conventional” volume) extends 5–6 mm above and below the CTV [17].

**Table 2:** OAR Constraints

	3D RT	SBRT 3 fractions	SBRT 5 fractions
**Spinal cord**	Dmax <45Gy	V18 ≤ 10% for « partial » volume	V23 ≤ 10%
	(standard fractionation)	V18 ≤ 0.35 cm^3^ for « conventional » volume	V23 ≤ 0.35 cm^3^
		V12 ≤ 1.2 cm^3^ for « conventional » volume	V14.5 ≤ 1.2 cm^3^
**Cauda equina**		V22.5 < 5 cm^3^	V30 < 5 cm^3^
		D2% < 24 Gy	D2% < 32 Gy
**Sacral plexus**		V22 < 3 cm^3^	V30 < 3 cm^3^

*3D RT* 3 Dimensional Radiation Therapy; *SBRT* Stereotactic Body radiation Therapy

For the others OAR not listed in Table 2, investigators must refer to AAPM Task Group 101 report. It is important to deliver as low a dose as possible in the organs at risk while assuring that a sufficient dose to the target volume is delivered.

#### Arm B

In the Control group (without SBRT), palliative radiotherapy on bone metastases is allowed if necessary (pain, fracture, spinal cord compression …).

The acceptable regimens for patients in Arm1 are as follows as per consensus guidelines [18]:

- 30 Gy / 10 fractions / 5 fractions per week
- 24 Gy / 6 fractions / 5 fractions per week
- 20 Gy / 5 fractions / 5 fractions per week
- 8 Gy / 1 fraction

The organs at risk are not delineated in the control group except for the spinal cord (Table 2).

### Ethical consideration and study registration

This Study has been approved by Nord-Ouest I Ethics Committee and French Regulatory authorities (ANSM). French ethics committee (CPP Nord-Ouest I) has approved this protocol on October 13th 2016 (reference number: CPP 02/016/2016). The study will be performed in accordance with the Declaration of Helsinki and will comply to the International Conference on Harmonization and Good Clinical Practice and General Data Protection Regulation (GDPR). This study funded by grants from French Cancer Institute (Institut National du Cancer, 52, avenue André Morizet, F-92513 Boulogne Billancourt Cedex) though PHRC program (grant number: PHRC-K 2015–149) that has no role in the collection, analysis, interpretation of results or writing of manuscripts. The study has been registered at clinicaltrials.gov (ClinicalTrials.gov NCT03143322 Registered on May 8th 2017).

Patient information and informed consent from the patient must be handled in accordance with the French regulation. Prior to the participation of a patient in the trial, this patient will be informed both verbally and in writing about the objectives of the trial, its methods, anticipated benefits and potential risks and the discomfort to which they may be exposed. The informed consent form for study and ancillaries studies, must be personally dated and signed by the patient and investigator.

### Quality Assurance of Radiation Therapy

In order to ensure patient safety and effective treatment delivery, a quality assurance protocol is implemented. Prior to opening the study, each participant centre will have to validate a dummy run by delineate targets and OARs and produce a treatment plan from a benchmark case on Aquilab software. The Quality Assurance committee will evaluate the dummy run. Patient inclusions by a centre will be allowed only after full validation. Afterwards, each participating centre will have to send planning and contouring information for the first included patient for review before the start of the radiotherapy. The QA committee according to the protocol recommendations in terms of delineation, dose constraints and treatment workflow will evaluate this treatment plan. At the end of treatment, each participating centre will have to send final review of the RT data for all included patients in SBRT arm for review. The QA committee, according to the protocol recommendations in terms of delineation, dose constraints and treatment workflow will evaluate the treatment plan for Quality Assurance.

### Systemic treatment

The patients will receive systemic standard of care for the metastases according to current oncological national recommendations and international guidelines.

### Follow-up

Patients will be seen at 3 months, 6 months post randomization and then every year for 3 years.

(Table 1). At each visit, the oncologist will conduct a history and physical examination, and assess CTC-AE toxicities, pain and quality of life. CT head, chest, abdomen and pelvis will be repeated at 6 months, then every year for 3 years. PET-CT and SPECT-CT will be repeated at 1 year. Additional Imaging should be carried out at the discretion of the physician. Additional treatment (e.g. further chemotherapy) is at the discretion of the oncologists.

### Measurement of response

Progression-free survival will be measured as time to either progression or death, whichever occurs first. Efficacy Evaluation will be performed at baseline, at 6 months, at 1 year, then subsequently every year until the end of study, using the same imaging method as for baseline (CT scan or MRI or PET if indicated).

Lesion response will be evaluated in this study using the RECIST criteria, PERCIST Criteria and MD Anderson Criteria [19].

### Statistical analysis

Sample size calculation: 196 patients will be randomized between 2 arms (in a 1:1 ratio). The primary endpoint is the 1-year Progression Free Survival (PFS). In the standard arm, the expected progression-free survival at 1 year is 30% (10% for lung cancer, 50% for breast cancer and 30% for prostatic cancers). In the experimental arm, we anticipate a 20% absolute increase (i.e. 30, 70 and 50%, respectively). According to Freedman’s method, accepting a two-tailed type I error of 5% and an 80% power (type II error of 20%), 178 patients (2 X 89) need to be recruited and 106 events need to be observed for the primary analysis. Assuming a 10% withdrawal rate, a total of 196 patients will need to be included.

Data analysis: All patients will be randomized after completion of the primary tumor treatment for synchronous or metachronous metastasis. The randomization will be stratified by date of metastasis (synchronous vs. metachronous) and primary tumor site (breast vs. non-small cell lung vs. prostate cancer). Statistical analysis will be performed on all the randomized patients, accordingly to the intent to treat (ITT) arm, whatever the actually received treatment. Patients without progression and alive at the time of analysis will be censored at the time of the latest assessment.

Primary Endpoint Analysis: Progression Free Survival PFS is defined as the time from randomization until the date of the first objective progression accordingly to RECIST or death (whatever its cause in the absence of progression). Patients without progression and alive at the time of analysis will be censored at the time of the latest assessment. The primary analysis will take place when 106 events have been observed. This analysis will use the logrank test, stratified on the primary location and the time of onset of the bone metastases (synchronous vs metachronous). Cox regression model will also be used to perform a sensitivity analysis taking into account the usual prognostic factors. The hazard ratio will be given with its 95% confidence interval. A *p* value <= 0.05 will be considered as statistically significant. A secondary analysis will also be performed, using the Fine and Gray model in order to take into account the competing risks (relapse in the bone, relapse in another site, death), and so to analyse the impact of the different PFS events on the trial results.

### Data safety monitoring committee

An Data Safety Monitoring Committee (DSMC), with expertise and experience in the pathology, and without direct involvement in the conduct of the trial, will be set up specifically to guarantee effective protection of patients, insure the ethical conduct of the trial, benefit/risk ratio of the trial, and to ensure the independent review of the scientific results during the trial and at the end of the trial. The DSMC will meet after 60 patients are accrued in the experimental arm to review toxicity outcomes and compliance.

The DSMC may recommend the early termination of the trial if one of the following conditions is met: an unacceptable toxicity or all available data from the trial or any other source of information are sufficiently convincing to influence the therapeutical practices of the majority of clinicians. The DSMC has only a consultative role; it will inform the sponsor who will decide whether the DSMC recommendation will be followed. An adverse event (AE) is defined as any untoward medical occurrence, in a patient or clinical trial subject treated by a medicinal product and which does not necessarily have a causal relationship with this treatment. The assessment of whether there is a reasonable causal relationship is made by the investigator. Notification must be carried out immediately by fax to the R&D UNICANCER pharmacovigilance unit by sending the form “notification of a Serious Adverse Effects”, located in the Investigator Master File, completed as precisely as possible, dated and signed by the physician-investigator.

### Biological ancillary study

The follow-up of bone metastasis evaluation is difficult by means of anatomic imaging (CT-scan). There is a need to identify biologic markers that would predict bone disease progression (DP) and risk of skeletal-related events (SREs) as sequelae of bone metastases. As this ancillary study, we have chosen to explore s-CTX and bone alkaline phosphatase (b-ALP) to better quantify response to SBRT vs standard treatment [20–22]. Predictive value of these biomarkers at different points will be assessed on disease control rate (DCR) [23–26]. To perform this study, blood samples (sCTX and bALP: 7 ml in a dry tube will be collected before the beginning of treatment, at the end of treatment, at 3 months, 6 months and 1 year after randomization.

### Economic ancillary study

Cost-utility, cost-effectiveness, and budget impact analyses performed on QALYs (Quality-Adjusted Life Years) and ICERs (Incremental Cost-Effectiveness Ratios) calculation based on EQ-5D-3L questionnaire will provide useful and complementary information in order (i) to recommend the best strategy to adopt; (ii) to estimate the budget impact on the French National Health Insurance of the generalization of the cost-effective strategy [27–29]. Analyses will be performed comparing SBRT versus conventional treatment without SBRT in solid tumors patients with ≤3 bone-only metastasis. Incremental costs between both arms will be compared to incremental health improvement.

## Discussion

Stereotactic radiotherapy is a routine treatment for brain, hepatic and lung metastases with increased probability of local control. However, Loco Regional Control is similar to bone metastasis than other localizations with 95% and a good control of pain and patients with oligometastasis had a longer survival with better local control for spine metastasis [10, 11].

The tools required for high precision radiotherapy are now commercially available and implemented in many radiotherapy departments. Sufficient clinical experience has been acquired so that large randomized trials can confidently be launched. Current knowledge is mainly based on retrospective and prospectives non-randomized studies, thus suffering from heterogeneous population and inappropriate sample power. The oligometastatic concept has been developed in the last years and recent trials show interesting results on adding Metastase Directed Treatment (MDT) (such as radiotherapy or surgery) at standard therapy. However, despite encouraging data, some authors remain skeptical that MDT will delay the use of systemic treament.

In a randomized phase II trial, named SABR-COMET, a benefit in OS in the SBRT arm for the oligometastatic setting compared to palliative treatment was demonstrated (41 months vs 28 months) [30]. In oligometastatic NSCLC, Gomez et al., that conducted a randomized phase II trial on oligometastatic NSCLC, reporting a significant PFS benefit with LCT (both radiotherapy or surgery) vs maintenance systemic therapy (14.4 months vs 4.4 months respectively). In oligometastic prostate cancer, HORRAD, STAMPEDE, STOMP and ORIOLE studies have shown encouraging results [31–34]. In oligometastatic breast cancer, Steven David et al. demonstrated that SABR is feasible, well tolerated and effective in selected patients with bone-only oligometastatic disease [35].

Two other randomized trials, SARON and CORE investigate the same approach in different clinical settings with oligometastatic patients [36]s. With respect to these two studies, Stereo-OS will allow a specific focus on skeletal metastases only and is thus expected to bring a clear-cut assessment of the added benefit of stereotactic radiotherapy on PFS in the especially dedicated treatment of a highly frequently occurring type of metastases in solid tumor cancers.

In a recent review of Faiez Al-Shafa et al., on Ongoing Trials of Stereotactic Ablative Radiotherapy for Oligometastatic Cancers, A minority of trials were randomized in design (*n* = 17, 27%). While most studies allowed for metastases from multiple primary disease sites (*n* = 22, 34%), the most common was prostate (*n* = 13, 15%), followed by breast, gastrointestinal, non-small cell lung cancer (NSCLC), and renal (*n* = 6, 9% each). In studies with a solitary target site, the most common was liver (*n* = 6, 9%) followed by lung (*n* = 3, 5%). The most common primary endpoints were progression-free survival (PFS) (*n* = 20, 31%) and toxicity (*n* = 10, 16%). A combined strategy of systemic therapy and SABR was an emerging theme (*n* = 23, 36%), with more recent studies specifically evaluating SABR and immunotherapy (*n* = 9, 14%) [37].

The safety and efficacy of SABR as oligometastasis-directed treatment is increasingly being evaluated within prospective clinical trials. These data are awaited to compliment the abundance of existing observational studies and to guide clinical decision-making.

## Data Availability

Data sharing is not applicable to this article as no datasets were yet generated or analyzed during this ongoing study.

## Abbreviations

SABR: Stereotactic ablative radiotherapy
WBRT: Whole brain radiotherapy
CTC-AE: Common Terminology Criteria for Adverse Events
CT: Computed tomography
PET: Positron emission tomography
MRI: Magnetic resonance imaging
GTV: Gross tumor volume
CTV: Clinical target volume
PTV: Planning target volume
PRV: Planning organ at risk volume
PI: Principal investigator
QA: Quality assurance
RECIST: Response evaluation criteria in solid tumors
PERCIST: Positron Emission Tomography (PET) Response Criteria in Solid Tumors
PFS: Progression Free Survival
OS: Overall survival
DSMC: Data Safety Monitoring Committee
ITT: Intent to treat
QALYs: Quality-Adjusted Life Years
ICERs: Incremental Cost-Effectiveness Ratios
QLQ: Quality of Life Questionnaire
Gy: Gray
FDG: Fluorodeoxyglucose
NA-F: Sodium Fluoride
EORTC: European Organisation for Research and Treatment of Cancer
ESMO: European Society for Medical Oncology
INCa: Insitut du Cancer
